# The Effectiveness of a Telenutrition Intervention to Improve Dietary Behavior and Physical Activity Among Adolescents With Obesity: Protocol for a Systematic Review

**DOI:** 10.2196/53282

**Published:** 2024-03-05

**Authors:** Muhammad Ridwan Ansari, Nurul Kodriati, Ariani Arista Putri Pertiwi, Fatwa Sari Tetra Dewi

**Affiliations:** 1 Faculty of Medicine, Public Health and Nursing Universitas Gadjah Mada Yogyakarta Indonesia; 2 Department of Nutrition Faculty of Public Health Universitas Ahmad Dahlan Yogyakarta Indonesia; 3 Departement of Public Health Faculty of Public Health Universitas Ahmad Dahlan Yogyakarta Indonesia; 4 Department of Basic Nursing and Emergency Faculty of Medicine, Public Health and Nursing Universitas Gadjah Mada Yogyakarta Indonesia; 5 Department of Health Behavior, Environment and Social Medicine Faculty of Medicine, Public Health and Nursing Universitas Gadjah Mada Yogyakarta Indonesia

**Keywords:** telehealth, obesity, telenutrition, adolescent, behavior change, virtual counseling, teenager, young adult, food intake, dietary pattern, intervention

## Abstract

**Background:**

The global obesity pandemic among adolescents is becoming a public health issue throughout the world. Telehealth use has significantly increased during and after the COVID-19 pandemic, including its application in adolescent obesity prevention and treatment.

**Objective:**

This review aims to synthesize the evidence on the effectiveness of telenutrition in improving dietary behavior and physical activity in adolescents with obesity.

**Methods:**

The PRISMA-P (Preferred Reporting Items for Systematic Reviews and Meta-Analysis Protocols) guideline will be used to structure this protocol. The focus of the systematic review is guided by the population, intervention, comparator, and outcome (PICO) framework. A systematic search of Science Direct, PubMed, Cochrane, Embase, JMIR, ProQuest, and Google scholar databases will be conducted. Two authors will screen the titles and abstracts of identified studies independently and select studies according to the eligibility criteria. The full-text reading will be done independently by 2 reviewers to assess final eligibility. Any discrepancies will then be discussed and resolved. The Cochrane Collaboration Risk of Bias tool was used to assess the risk of bias; a descriptive analysis will summarize the effectiveness of the telenutrition or any type of telehealth intervention used.

**Results:**

The systematic review is expected to be completed by the end of March 2024. The ongoing screening and review of the articles are currently being conducted.

**Conclusions:**

This systematic review aims to summarize the effectiveness, features, design process, usability, and coherence of a telenutrition intervention using behavior change theory to improve dietary patterns and physical activity among adolescents with obesity. It will identify areas for improvement and best practices, informing the development of more useful and engaging telenutrition interventions for adolescents.

**Trial Registration:**

PROSPERO CRD42023458336; http://tinyurl.com/cp46fjj9

**International Registered Report Identifier (IRRID):**

DERR1-10.2196/53282

## Introduction

The global obesity pandemic is becoming a public health issue, particularly among children and adolescents aged 5 to 19 years [1]. Between 2013 and 2018, the trend of adolescent obesity nearly doubled in Indonesia [2,3]. Obesity is caused by unhealthy lifestyles, such as most adolescents not meeting the daily fruit and vegetable recommendation and engaging in less physical activity; additionally, approximately 65% of adolescents skip breakfast, and 56% of adolescents aged 15-19 years have regular daily consumption of sugar-sweetened beverages [4,5].

To address this issue, various programs and interventions have been implemented. Nutrition literacy interventions combined with behavior change communication interventions in a school-based setting have shown to be promising for improving BMI and reducing unhealthy food choices. The school environment has been identified as a strategic channel for health promotion among school-age adolescents [6,7]. However, certain gaps have been identified that explain why health and nutrition programs in school settings in Indonesia have not been as effective as desired. Some schools encountered competing priorities in supporting the program, such as a lack of teachers and time due to teaching constraints. Additionally, 21% of adolescents were not going to school, compounded by insufficient parental support [8,9].

Telehealth is an alternative method for increasing access to adolescents, as the target population, and their parents. A previous systematic review revealed that there is great potential in digital platforms for universal health promotion, especially among school-age children and adolescents [10]. Digital nutrition literacy combined with a behaviour change program delivered via a telehealth platform could be a promising solution to bridge the gap. The International Organization for Standardization defines telehealth intervention for children as “the use of telecommunication techniques for the purpose of providing telemedicine, medical education, and health education over a distance” [11]. A review was carried out to assess the use of digital health interventions, such as websites, short-text messages, gamification, social media, and multidigital component interventions, aimed at improving adolescent diet and physical activity. The review revealed that website interventions can influence behavior change in adolescent diet and physical activity. However, due to variability in engagement, these changes often are not sustained in the medium or long term [12]. Meanwhile, there is less evidence supporting the effectiveness of other digital platforms, such as apps, text messages, and social media [12].

During and after the COVID-19 pandemic, there has been an increase in evidence supporting the impact of telehealth interventions in improving health [13,14]. This medium provides more engagement features compared to other forms of digital intervention. Therefore, a systematic review is required to address the following:

- Synthesize the evidence on the effectiveness of telenutrition in improving dietary behavior and physical activity in adolescents with obesity.
- Identify specific intervention components that characterize the more successful telenutrition intervention, particularly in adolescent populations.

## Methods

### Overview

We will use the population, intervention, comparator, and outcome (PICO) framework to identify appropriate Medical Subject Headings (MeSH) terms for the literature search. Meanwhile, the PRISMA-P (Preferred Reporting Items for Systematic Reviews and Meta-Analysis Protocols) 2015 guideline will be used as the standard reporting protocol guideline checklist. The PRISMA-P 2015 guideline will aid in describing the rationale, hypothesis, and planned methods of the review [15]. The checklist is provided in Multimedia Appendix 1.

This systematic review will comprise a literature search, article selection, data extraction, quality appraisal, data analysis, and data synthesis. The protocol of this systematic review was prospectively registered on PROSPERO (CRD42023458336).

### Eligibility Criteria

The PICO framework presented in Table 1 is derived from the research questions previously mentioned.

**Table 1:** The eligibility criteria based on the population, intervention, comparator, and outcome (PICO) framework.

Criteria	Detailed information
Population	Adolescents or young adults aged 13-18 years with overweight or obesity.
Intervention	Any digital health promotion intervention with any type of telehealth (including but not limited to either educational or personalized feedback or monitoring services).
Comparator	Other digital health promotion interventions delivered by other methods, such as social media, websites, and short-text messengers or studies with control groups without an intervention.
Outcomes	Healthy behavior and nutritional outcome, such as changes in dietary behavior, physical activity level, and BMI.

### Search Strategy

We will search the following databases: Science Direct, PubMed, Cochrane, Embase, JMIR, ProQuest, and Google Scholar. Key terms relating to telehealth and telenutrition were extracted from an initial review of the literature. Specific search terms, such as “obesity,” “adolescent,” “dietary behaviors,” and “physical activity,” were identified in a preliminary scan of the literature and chosen in consultation with a medical librarian. Search terms will include MeSH terms and related keywords and are grouped into 3 themes presented in Table 2. All the MeSH terms and Keywords are included in Table 2 with the following structure: Obesity (MeSH OR keywords) AND Adolescent (MeSH OR keywords) AND Telenutrition (MeSH OR keywords). To broaden the scope of the article collection, we will not limit the publication period or language of publication in our search strategy. In addition to exploring these databases, a manual search will be performed to identify the reference lists of included studies. Authors of conference and poster abstracts selected for inclusion will be contacted to see if a full text is available to be included.

**Table 2:** Search terms.

Number	Category	MeSH^a^ terms and keywords in titles or abstracts
1	Obesity	“Obesity“[Mesh] OR “Pediatric Obesity”[Mesh] OR “overweight”[MeSH Terms] OR “overweight”[All Fields]
2	Adolescent	(“Adolescent” OR “Adolescent Behavior” OR “Adolescent Health” OR adolesce*)
3	Telenutrition	(“Telenutrition” OR “Mobile Health” OR “mHealth” OR “Telehealth” OR “eHealth” OR “Short Message Service” OR “SMS” OR “Text Messag*” OR “cell phone” OR “telephone” OR “smartphone” OR “cellular” OR “mobile” OR “social media” OR “social network”)
4	All categories	1 AND 2 AND 3

^a^MeSH: Medical Subject Headings.

### Inclusion Criteria

Only human intervention studies, either quasi-experimental studies or randomized controlled trials, will be included in this review. This review will evaluate original research articles using digital health promotion interventions delivered via any type of telehealth and involving adolescents aged 13-18 years with overweight or obesity. Overweight and obesity among adolescents aged 5-19 years are defined by their BMI per age *z* score, as outlined in the World Health Organization growth chart references [16]. We will include any study that categorizes participants with a BMI per age *z* score >1 SD as overweight and those with a BMI >2 SD as obese.

### Exclusion Criteria

The review focused mainly on the changes in healthy behaviors and nutritional outcomes, including dietary behavior, physical activity levels, and BMI. Any outcomes other than these will be excluded from our review. In addition, articles for which full texts cannot be obtained despite requests to the corresponding authors will be excluded.

### Screening and Article Selection

All articles found through database searches will be saved in the citation and article management software Mendeley and Rayyan AI, which will be used to eliminate duplicates and screening. The titles and abstracts of all studies will be reviewed independently by 2 reviewers. Studies that do not meet the eligibility criteria will be excluded, and any disagreements will be discussed until a consensus is reached. The full text of the remaining studies will then be examined by 2 reviewers independently to determine final eligibility, with any disagreements resolved by a third and fourth reviewer. A PRISMA flow diagram will be used to record the details of the screening and selection process so that the study can be replicated.

### Data Extraction and Management

One reviewer will read the full text of all the papers included in the final selection to extract the predetermined outcomes, which will be validated by a second reviewer. The outcomes will be extracted into a standard extraction form, which will be designed in Covidence (Veritas Health Innovation). The detailed extraction custom-built form is summarized in Table 3. Disagreements will be resolved through discussion, and if consensus cannot be reached, a third and fourth reviewer will be consulted. Missing data will be considered in the risk of bias assessment, but due to time constraints, authors will not be contacted.

**Table 3:** Summary of the data extraction template.

Information	Detail extraction
General study information	Year of publication Study setting (including the time frame before or after the COVID-19 pandemic, if available) Analyzing the sample size Sample demographics (including age, gender, and target population) Intervention design information (including type, duration, and follow-up periods, if any)
Behavioral and nutrition literacy intervention	Target health behaviors and intervention focus Theory of the intervention Behavior change technique
Telehealth and telenutrition	Area of health care used Name of the platform Developers Media platform Design process or steps Component and design features (ie, gamification, families’ involvement, and SMS reminder)
Evaluation	The outcomes measured Any kind of effectiveness measurement Participant health outcomes Participant engagement or adherence rates (including participation or drop-out rates) Feasibility and usability Challenges and limitations (including ethical concerns and barriers during the experiment)

### Quality Appraisal and Risk of Bias Assessment

Following the final selection of studies, 2 reviewers will independently assess the risk of bias in all papers included in the final selection. If there is a disagreement in judgment, the reviewers will discuss it before consulting the third and fourth reviewers. The Cochrane Collaboration Risk of Bias tool will be used to assess the randomized controlled trials included in the review and assign low, unclear, or high risk to the studies for each of the potential biases [17]. Studies will be classified as low risk if the identified bias is unlikely to significantly alter the results, and they are classified as high risk if the bias clearly has the potential to significantly alter the results. Unclear risk occurs when the bias imposes some doubt on the outcome. The risk of bias assessment will cover 6 domains: selection, performance, detection, attrition, reporting, and other bias [17].

Meanwhile, ROBINS-I (Risk Of Bias In Non-randomized Studies of Interventions) will be used for evaluating the risk of bias in estimates of the comparative effectiveness of interventions from studies that did not use randomization to allocate units to comparison groups [18]. A table will be created that summarizes the quality of all included studies.

### Data Analysis and Synthesis

A meta-analysis is unlikely to be conducted due to the expected diversity of study aims, methods, and reported outcomes. As a result, we will conduct a descriptive analysis to summarize the extracted data. Each outcome will be coded as having no (0), some (+), or significant evidence (++) of effectiveness in both outcomes. Significant evidence will be coded only when the intervention (ie, nutritional literacy and behavior change communication via telehealth delivery) outperforms a comparator or control. The intervention will be considered to have some evidence of effectiveness if there is a significant difference over time but not between groups or a significant improvement in only a subgroup of the population. Studies will be grouped by target health behavior (eg, dietary pattern only, physical activity only, or both) and analyzed together to describe the effectiveness of telehealth or telenutrition interventions in general for both those target behaviors; in particular, the analysis will focus on either dietary pattern or physical activity. The discussion will synthesize the data to describe specific intervention components that characterize a successful telenutrition intervention, such as the type of digital platform, engagement features, theory, method, and behavior change strategy used. The theory, method, and behavior change strategy will be analyzed and presented based on the intervention mapping approach and its taxonomy, as described by Bartholomew et al [19] and Kok et al [20]. Furthermore, the information regarding the identified barriers and challenges of telehealth interventions will be summarized to give directions for future research and development.

### Ethical Considerations

There will be no involvement from patients or the public in this study. This study synthesizes data from studies that were already peer reviewed and published. Thus, no ethical approval will be required.

## Results

The systematic review is expected to be completed by the end of March 2024. The ongoing review and data extraction of the articles is currently being conducted. The findings of this study will be outlined in the following subsections: (1) study selection, (2) study characteristics, (3) synthesis of results (behavior change strategy and method or telehealth development and intervention), as well as (4) impact and evaluation, including challenges and barriers of study implementation.

## Discussion

A systematic and transparent review of the literature will provide a better understanding of the current state-of-the-art telehealth or telenutrition approaches, how they are used, and their effectiveness. Strengths, limitations, and implications for the interaction of technology and behavioral health management will help inform and improve the development, acceptability, and effectiveness of future telehealth approaches in managing adolescent obesity for clinicians, telehealth providers, and relevant stakeholders. Based on the data, we will outline the conclusions, the strengths and limitations of our systematic review, and important directions for future research. Telehealth has grown over the past decade, with its popularity increasing during the COVID-19 pandemic. In addition, our review aims to differentiate findings between before and after the COVID-19 pandemic, if possible. Furthermore, the findings will be interpreted carefully, taking into consideration the potential scalability to other areas or countries with similar contexts. Our study has some potential limitations due to limited resources; we were unable to conduct manual searching and identification of reference lists of included studies. The review also could not provide a clear effect estimate between the telehealth intervention and health outcomes, as a meta-analysis could not be conducted.

## Appendix

Multimedia Appendix 1 PRISMA-P (Preferred Reporting Items for Systematic Reviews and Meta-Analysis Protocols) checklist.

## Abbreviations

MeSH: Medical Subject Heading
PICO: population, intervention, comparator, and outcome
PRISMA-P: Preferred Reporting Items for Systematic Reviews and Meta-Analysis Protocols
ROBINS-I: Risk Of Bias In Non-randomized Studies of Interventions
